# The Fur regulon in anaerobically grown *Salmonella enterica sv*. Typhimurium: identification of new Fur targets

**DOI:** 10.1186/1471-2180-11-236

**Published:** 2011-10-21

**Authors:** Bryan Troxell, Ryan C Fink, Steffen Porwollik, Michael McClelland, Hosni M Hassan

**Affiliations:** 1 Department of Microbiology, North Carolina State University, Raleigh, NC 27695-7615, USA; 2 The Vaccine Research Institute of San Diego, 10835 Road to the Cure, Suite 105, San Diego, CA 92121, USA; 3 Department of Immunology and Microbiology, Indiana University School of Medicine, Indianapolis, IN 46202, USA; 4 Department of Food Science and Nutrition, University of Minnesota, St. Paul, MN 55108-1038, USA

## Abstract

**Background:**

The Ferric uptake regulator (Fur) is a transcriptional regulator that controls iron homeostasis in bacteria. Although the regulatory role of Fur in *Escherichia coli *is well characterized, most of the studies were conducted under routine culture conditions, i.e., in ambient oxygen concentration. To reveal potentially novel aspects of the Fur regulon in *Salmonella enterica *serovar Typhimurium under oxygen conditions similar to that encountered in the host, we compared the transcriptional profiles of the virulent wild-type strain (ATCC 14028s) and its isogenic Δ*fur *strain under anaerobic conditions.

**Results:**

Microarray analysis of anaerobically grown Δ*fur S*. Typhimurium identified 298 differentially expressed genes. Expression of several genes controlled by Fnr and NsrR appeared to be also dependent on Fur. Furthermore, Fur was required for the activity of the cytoplasmic superoxide disumutases (MnSOD and FeSOD). The regulation of FeSOD gene, *sodB*, occurred via small RNAs (i.e., the *ryhB *homologs, *rfrA *and *rfrB*) with the aid of the RNA chaperone Hfq. The transcription of *sodA *was increased in Δ*fur; *however, the enzyme was inactive due to the incorporation of iron instead of manganese in SodA. Additionally, in Δ*fur*, the expression of the gene coding for the ferritin-like protein (*ftnB*) was down-regulated, while the transcription of the gene coding for the nitric oxide (NO^·^) detoxifying flavohemoglobin (*hmpA*) was up-regulated. The promoters of *ftnB *and *hmpA *do not contain recognized Fur binding motifs, which indicated their probable indirect regulation by Fur. However, Fur activation of *ftnB *was independent of Fnr. In addition, the expression of the gene coding for the histone-like protein, H-NS (*hns*) was increased in Δ*fur*. This may explain the observed down-regulation of the *tdc *operon, responsible for the anaerobic degradation of threonine, and *ftnB *in Δ*fur*.

**Conclusions:**

This study determined that Fur is a positive factor in *ftnB *regulation, while serving to repress the expression of *hmpA*. Furthermore, Fur is required for the proper expression and activation of the antioxidant enzymes, FeSOD and MnSOD. Finally, this work identified twenty-six new targets of Fur regulation, and demonstrates that H-NS repressed genes are down-regulated in Δ*fur*.

## Background

The Ferric uptake regulator (Fur) is a metal-dependent regulator of transcription and post-transcription in bacteria, which senses metal concentration and/or the redox state of the cells (reviewed in [1]). The classical model of the regulatory role of Fur depicts transcriptional repression through ferrous iron that results in Fur-Fe^2+ ^binding to the operator site of a target gene [2,3]. Fur-Fe^2+ ^binding to DNA are presumed to be homodimeric; however, multimeric complexes have been reported [4,5]. In addition, the metal cofactor present *in vivo *is controversial, due to the ability of the Fur protein to bind different divalent cations, *in vitro *[6]. For example, Fur represses aerobactin biosynthesis using ferrous iron, cobalt, or manganese [2]. Moreover, most researchers studying Fur binding to promoter sequences, *in vitro*, employ manganese instead of ferrous iron due to the reactivity of ferrous iron with oxygen. However, evidence exists that Fur regulates specific genes differently in the presence of ferrous iron or manganese [7]. Fur also contains zinc for protein stability [8,9]. This indicates that the availability of the metal cofactor to pathogens residing in the host dictates the activity of Fur.

The regulatory role of Fur has been demonstrated in numerous pathogenic and non-pathogenic organisms growing in the presence of ambient oxygen [10-19]. However, research has shown that the oxygen concentration in the host is low. For example, the oxygen sensitive [20], Fnr (Fumarate nitrate reduction) was shown to be essential for virulence in *Salmonella enterica *serovar Typhimurium (*S*. Typhimurium) [21], *Shigella flexnari *[22], *Neisseria meningitidis *[23], and *Pseudomonas aeruginosa *[24]. In addition, the expression of the dimeric Cu-Zn superoxide dismutase (SodCI), one of the virulence determinants in *S*. Typhimurium, within the J774.1 cell line was shown to be Fnr-dependent [25]. Fnr is a transcriptional regulator that is active as a homodimer and contains an oxygen labile iron sulfur cluster (4Fe-4S) [26]. Fnr can serve either as an activator or as a repressor of transcription, depending on the target gene. For instance, under anaerobic conditions, Fnr represses the cytochrome *c *oxidase (*cyoABCDE*) and the cytochrome *bd *complex (*cydAB*), while activating genes important for utilizing alternative electron acceptors such as fumarate [21]. Therefore, it is reasonable to conclude that O_2 _concentration within the host is low enough to activate Fnr in *S*. Typhimurium residing within cells of the innate immune system. This *in vivo *low oxygen concentration appears to be sufficient to cause a shift in the redox state of iron from ferric to ferrous. Indeed, when *S*. Typhimurium is within macrophages, repression of the Fur regulated *iroBCDE *promoter occurs regardless of the presence of the host metal transporter Nramp1 [27,28]. This demonstrates that during intracellular growth of *S*. Typhimurium, the state of oxygen tension and iron valence are adequate for the activation of both Fnr and Fur, respectively. Recently, we demonstrated the role of Fur in HilA expression and virulence in *S*. Typhimurium, which is mediated by the negative regulation of H-NS by Fur under anaerobic conditions [29].

H-NS is a DNA binding protein that is associated with the nucleoid of Gram-negative enteric bacteria (reviewed in [30]). Deletion of *hns *is considered lethal unless an additional mutation occurs in either the alternative sigma factor, *rpoS*, or the transcription factor, *phoP *[31]. H-NS binding can alter the topology of DNA and influence gene regulation [32]. Typically, H-NS exhibits a repressive role in gene regulation, especially of genetic loci associated with virulence [31,33-35]. H-NS preferentially binds to AT rich segments of DNA, which are characteristic of horizontally acquired *Salmonella *pathogenicity islands (SPIs) [36]. Interestingly, H-NS also represses genes associated with anaerobic metabolism including those responsible for the degradation of L-threonine, encoded by the *tdc *operon, and are induced under anaerobic conditions [37]. H-NS binds the *tdc *locus and represses its transcription [31], thereby linking amino acid catabolism with H-NS regulation. In addition, Fur is known to activate SPI1 via the activation of the positive regulators of SPI1 (i.e., HilA and HilD) [38,39]. This activation is, in part, indirect where Fur represses the expression of *hns*, which represses the expression of *hilA *and *hilD *[29]. Thus, Fur indirectly activates SPI1 via its repression of *hns*, demonstrating that iron metabolism can influence genes regulated by H-NS.

Our goal here was to compare the transcriptome of wild-type (WT) *S*. Typhimurium to an isogenic strain lacking the *fur *gene (Δ*fur*) in cells growing under anaerobic conditions (i.e., conditions resembling that encountered by the pathogen during infection [40]). To accomplish that goal, we used DNA microarray analysis and operon reporter fusions. We found that Fur directly or indirectly regulates 298 genes (~6.5% of the genome); of these, 49 contained a putative Fur binding site. Interestingly, Fnr controls 15 of these 49 genes [21] and 12 of the 15 genes contain putative binding sites for both Fur and Fnr. This suggests a regulatory link between oxygen and iron availability through the action of these two global regulators, Fur and Fnr. Furthermore, Fur was required for the activity of both cytoplasmic superoxide dismutases (MnSOD and FeSOD). We also found that the anaerobic expression of *ftnB *(encoding a ferritin-like protein) and *hmpA *(encoding the NO· detoxifying flavohemoglobin) was dependent on both Fur and Fnr. However, the promoters of *ftnB *and *hmpA *do not contain recognizable Fur binding motifs indicating their indirect regulation by Fur. Increased expression of H-NS, a known repressor of *ftnB*, *tdc *operon, and other genes, in Δ*fur *may account for their activation by Fur. Finally, we have also identified twenty-six genes as new targets of Fur regulation in *S*. Typhimurium.

## Methods

### Bacterial strains, plasmids, growth conditions, and reagents

*S*. Typhimurium (ATCC 14028s) was used throughout this study, and for the constructing gene knockouts. Bacterial strains and plasmids used are listed in Table 1. Primers used were purchased from Integrated DNA Technologies (Coralville, IA) and are listed (Additional file 1: Table S1).

**Table 1:** Bacterial Strains and Plasmids

Strains	Genotype	Reference/Source
*Salmonella enterica *Typhimurium 14028s	'wild-type'	American Type Culture Collection
KLM001	*Δfur::bla*	[79]
NC 997	*Δfnr::cat*	This work
NC 1006	*Δfur::bla Δfnr::cat*	This work
NC1016	*Δhfq::FRT*	[29]
NC 1067	*ftnB'::lacZY*	This work
AV0305	*hmpA'::lacZY*	[125]
NC 1065	*Δfur::bla ftnB'::lacZY*	This work
NC 1066	*Δfur::bla hmpA'::lacZY*	This work
NC 1068	*Δfnr::cat hmpA'::lacZY*	This work
NC 1069	*Δfur::bla Δfnr::cat hmpA'::lacZY*	This work
NC 1077	*Δfnr::cat ftnB'::lacZY*	This work
NC1078	*Δfur::bla Δfnr::cat ftnB'::lacZY*	This work
NC1020	*Δfur::bla Δhfq::FRT*	This work

**Plasmids**		
pKD46	Phage *λ gam-bet-exo *under P*_araB_*	[41]
pCP20	*bla cat cI857 λPR flp *pSC101 oriTS	
pCE36	*ahp *FRT *lacZY+ *oriR6K	[46]
pKD3	*bla *FRT *ahp *FRT PS1 PS2 oriR6K	[41]
pKD4	*bla *FRT *cat *FRT PS1 PS2 oriR6K	[41]
pKD13	*bla *FRT *ahp *FRT PS1 PS4 oriR6K	[41]

All knockouts were constructed using λ Red mediated methodologies in the host strain carrying pKD46. The cells were grown in Luria-Bertani (LB) medium to an optical density (OD_600_) of 0.3 at which point 50 mM arabinose was added for 90 min [41]. The culture was centrifuged, electroporated with 1 μg of purified PCR product of the gene of interest, recovered in SOC media (20 g tryptone, 5 g yeast extract, 0.5 g NaCl, per liter plus 20 mM glucose) for 3 h, plated on LB agar with the appropriate antibiotic, and incubated at 37°C. Transformants were verified by PCR followed by DNA sequencing. P22 phage transduction was used to move the mutations into the specified genetic backgrounds of *S*. Typhimurium 14028s. Colony PCR was used to confirm the genotype(s). Transductants were purified on Evans-Blue-Uranine (EBU) agar plates.

The medium used throughout this study was a buffered (pH = 7.4) LB containing 100 mM MOPS and 20 mM xylose (LB-MOPS-X) [21,29,42,43]; where indicated, kanamycin and ampicillin were used at 55 μg ml^-1 ^and 100 μg ml^-1^, respectively. Anaerobic conditions were maintained in a Coy anaerobic chamber (Coy Laboratory Products, Grass Lake, MI) filled with anaerobic gas mixture (10% H_2_, 5% CO_2_, and 85% N_2_). Media were equilibrated in the anaerobic chamber for at least 48 h prior to use. Aerobic conditions were maintained by shaking at 200 RPM at 37°C in a New Brunswick gyratory water bath. Growth was determined by measuring changes in OD_600 _over time. The ferrous iron chelator, 2, 2' dipyridyl (dip), was purchased from Sigma-Aldrich (St. Louis, MO) and used at 200 μM. PCR reagents were from Promega (Madison, WI).

### RNA isolation

For the microarray experiments, independent anaerobic cultures of 14028s and Δ*fur *(KLM001) were used to inoculate three independent flasks (150 ml of anoxic LB-MOPS-X) for each strain. The three independent cultures of 14028s and Δ*fur *were grown to an OD_600 _of 0.30 to 0.35 (~ four generations) and treated with RNAlater (Qiagen) to fix the cells and preserve the quality of the RNA as described previously [21,43]. Total RNA was extracted and its quality was assured before aliquots of the RNA samples were stored at -80°C for use in the microarray as previously described [21,43].

### Microarray studies

Serovar Typhimurium microarray slides were prepared and used as previously described [21,43,44]. The SuperScript Indirect cDNA labeling system (Invitrogen, Carlsbad, CA) was used to synthesize the cDNA for the hybridizations. Each experiment consisted of two hybridizations, on two slides carried-out at 42°C overnight. Dye swapping was performed to avoid dye-associated effects on cDNA synthesis. The slides were washed at increasing stringencies and the microarrays were scanned for the Cy3 and Cy5 fluorescent signals with a ScanArray 4000 microarray scanner from GSI Lumonics (Watertown, MA). The intensity of each spot was expressed as the sum of the intensities of the pixels included in a circle positioned over the spot. The background was the sum of the intensities of an identical number of pixels surrounding the circled spot.

### Data analysis

Values of Cy3 and Cy5 for each spot were normalized over the total intensity for each dye to account for differences in total intensity between the scanned images. The data from the microarray analysis were evaluated by two methods as previously described [21,43]. Briefly, the data were evaluated by a pair-wise comparison, calculated with a two-tailed Student's t test and analyzed by the MEAN and TTEST procedures of SAS-STAT statistical software (SAS Institute, Cary, NC) the degrees of freedom for the t test were calculated as described previously [21,43]. The t statistic was performed using the, two-tailed, heteroscedastic TTEST function of Excel software (Microsoft Corporation, Redmond, WA). The signal intensity at each spot from Δ*fur *and the WT was analyzed and used to calculate median expression ratios and standard deviations for ORFs showing at least 2.5-fold change and p < 0.05 [21,43].

### Microarray data

The microarray data are accessible via GEO accession number GSE18441 at http://www.ncbi.nlm.nih.gov/geo/query/acc.cgi?acc=GSE18441.

### Logo graph and promoter analysis

The information matrix for the generation of the Fur logo was produced using the alignment of the *Escherichia coli *Fur binding sequences, available at http://arep.med.harvard.edu/ecoli_matrices/. To account for slight variation in nucleotide usage between *E. coli *and *Salmonella*, a second alignment for *S*. Typhimurium was built using the 5^' ^regions of the homologous genes used to build the *E. coli *information matrix. The new alignment was used to generate an information matrix specific for *S*. Typhimurium. A graphical representation of the matrix through a logo graph was obtained with Weblogo software (version 2.8.1, 18 October 2004), available at http://weblogo.berkeley.edu. The information matrix was used to scan the 5' region (from the position -400 to +50) of the genes with significant variations of transcripts using the Patser software (version 3d), available at http://rsat.ulb.ac.be/rsat/.

If a sequence corresponding to a Fur binding motif was identified, then this sequence was given a weighted score [45].

### Construction of transcriptional *lacZ *fusions

Single-copy genomic transcriptional *lacZ *fusions were constructed as described previously [46]. Briefly, 300 ng of pCP20 was transformed into mutant strains; cultures were transferred twice at 30°C, and checked for loss of the antibiotic marker. Plasmids with a single FRT site upstream of promoterless *lacZY *were transformed into mutant strains carrying pCP20 and incubated at 37°C on an LB-agar plate with kanamycin. Transformants were transferred three times at 40°C, verified by PCR, and transduced into appropriate background(s).

### β-galactosidase assay and "Differential Plot" presentation of the data

The β-galactosidase assay was used to assess expression of transcriptional fusions in cultures growing at steady state. This was accomplished by 50-fold dilution of anaerobically grown overnight (~17 hr) cultures into fresh medium and once a steady state of growth was established, the cells were re-inoculated into fresh LB-MOPS-X medium to an OD_600 _~0.02. β-galactosidase assays were conducted during growth and the activity (U/ml) [47] was plotted against changes in OD_600 _in the form of a differential plot [48,49]; which are usually recommended for determining the rate of synthesis of an mRNA or a protein relative to the total rate of synthesis in the cell. The slope of the linear regression of this type of plot represents the differential rate of synthesis (i.e., Specific Activity, Units/OD_600_) during the steady state of growth. The intrinsic advantages of using this method (i.e., differential rate) over the commonly used method (i.e., one-time point assays) are well documented [50-53]. Data shown were from three independent cultures with standard deviation.

### Preparation of cell-free extracts and SOD activity gels

Cultures were grown anaerobically overnight, diluted to ~0.02 OD_600 _in LB-MOPS-X, and cells were harvested at OD_600 _~0.25. Further cell growth and *de novo *protein synthesis were minimized by adding chloramphenicol (50 μg ml^-1^) and ice to the cultures. In addition, 50 μg ml^-1 ^chloramphenicol was included at each step of sample preparation and handling. The cultures were sealed anaerobically and the cells collected by centrifugation at 5,000 × g at 4°C. Cells were washed with phosphate buffer (pH 7.8, 50 mM potassium phosphate containing 0.1 mM EDTA, KPi), centrifuged again, and resuspended in the same buffer. Cells were sonicated on ice for 15 sec on and 30 sec off for 15 min of total sonication time. Cell debris was cleared by centrifugation at 19,000 × g for 30 min at 4°C, and the supernatant was dialyzed against KPi in dialysis membranes with an 8,000 molecular weight cut-off. Dialyzed cell-free extracts were centrifuged at 20,000 × g for 30 min at 4°C, and the supernatant was stored at -80°C until use. Protein concentration was determined by the Lowry method [54]. Superoxide dismutase activity gels were performed using native 10% acrylamide gels as described previously [55].

### Fumarate reductase activity

Fumarate reductase activity (FRD) was assayed from cell-free extracts as described previously [56]. Briefly, cells were grown, cell-free extracts were prepared as described above, and the fumarate dependent oxidation of reduced benzyl viologen was determined. Specific activity of FRD is expressed as μmole of reduced benzyl viologen oxidized per minute per milligram of total protein.

### Measurements of total [Mn]

Independent anaerobic cultures were diluted to OD_600 _~0.02 and grown until OD_600 _0.35 in a Coy anaerobic chamber. Chloramphenicol was added at 50 μg ml^-1^, samples were sealed anaerobically, and centrifuged at 12,000 × g for 20 min at 4°C. Samples were washed with KPi as above, centrifuged, and resuspended in 2 ml of buffer. Samples were dried and treated with 3 M nitric acid overnight at room temperature then quickly boiled. Total manganese content was determined by Inductively Coupled Plasma Optical Emission Spectrometry (ICP-OES) at North Carolina State University Analytical Service Laboratory. Total manganese and iron was measured in LB medium as above using a 5X concentration of medium.

## Results

### Growth of Δ*fur *under anaerobic and aerobic conditions

**Figure 1:**
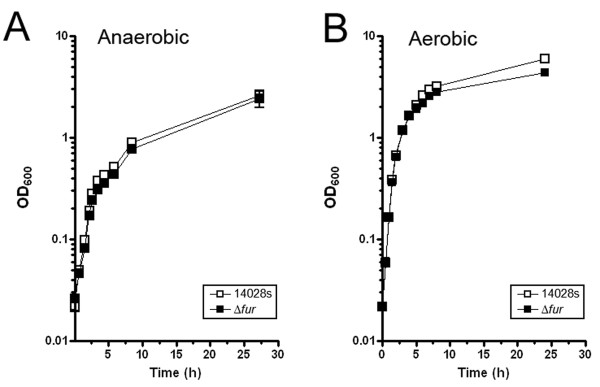
**Growth kinetics of Δ*fur *(black square compared to 14028s (white square)**. Cells were grown in LB-MOPS-X medium as described in Methods; **(A) **Anaerobic growth; **(B) **Aerobic growth.

Iron is an essential element for redox reactions in biology. However, it is an important factor in oxygen toxicity due to its involvement in hydroxyl radicals (HO·) formation via Fenton chemistry [57]. Therefore, we compared the effects of a deletion of *fur *on growth kinetics under both anaerobic and aerobic conditions. Data in Figure 1 demonstrate that Δ*fur *was not compromised in its growth kinetics under either anaerobic or aerobic conditions.

### Effect of Fur on the anaerobic transcriptome of *S*. Typhimurium

Under anaerobic conditions, the absence of *fur *resulted in the differential expression of 298 genes (Additional File 2: Table S2). These genes were organized by Cluster of Orthologous Groups (COGs) and the numbers of genes within each COG are shown in Table 2. The absence of *fur *resulted in increased expression (i.e., Fur acted as a repressor) of 226 genes. However, the absence of Fur resulted in decreased expression (i.e., Fur acted as an activator) of 72 genes, most likely via an indirect mechanism.

**Table 2:** Number of Differentially Expressed Genes in Δ*fur*

Differentially Expressed Genes in Δ*fur*				
**Cluster of Orthologous** **Groups**	**Number of** **Genes** **"Fur** **Repressed"^a^**		**Number of** **Genes** ** "Fur** **Activated"^b^**	**Total**

No COG	30		9	39
Energy Production and Conversion	16		18	34
Cell Cycle Control	3		0	3
Amino Acid Metabolism and Transport	7		16	23
Nucleotide Metabolism and Transport	7		4	11
Carbohydrate Metabolism and Transport	9		4	13
Coenzyme Metabolism and Transport	6		0	6
Lipid Metabolism and Transport	5		0	5
Translation	46		0	46
Transcription	9		2	11
Replication, Recombination, and Repair	5		1	6
Cell Wall/Membrane/Envelope Biogenesis	14		3	17
Cell Motility	1		0	1
Post-Translational Modification, Protein Turnover, Chaperone Functions	10		1	11
Inorganic Ion Transport and Metabolism	20		2	22
Secondary Metabolite Biosynthesis, Transport, and Catabolism	5		4	9
General Functional Prediction Only	15		4	19
Function Unknown	9		2	11
Signal Transduction Mechanisms	5		2	7
Intracellular Trafficking and Secretion	3		0	3
Defense Mechanisms	1		0	1

Total	226		72	298

Categorized According to Cluster of Orthologous Groups (COGs)

^a ^Genes with increased expression in the absence of *fur*

^b ^Genes with decreased expression in the absence of *fur*

**Figure 2:**
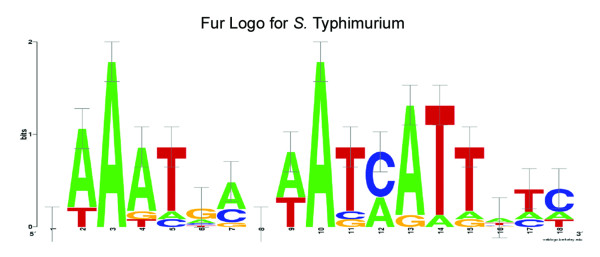
**Logo graph of the information matrix from the alignment of Fur-regulated genes in *S***. Typhimurium. The height of each column of characters represents information, measured in bits, for that specific position and the height of each individual character represents the frequency of each nucleotide.

A Fur information matrix, specific for *S*. Typhimurium, was generated (Figure 2), and used to scan the upstream regions of the 298 genes identified as differentially expressed in response to deletion of *fur*. We identified 49 genes that contain a putative Fur binding site (Table 3 - columns 1 & 2 and Additional file 2: Table S2).

**Table 3:** Newly Identified Genes Regulated by Fur That Contain a Predicted Fur Binding Site

Gene	Function	Fold Change^a^	Predicted Fur Binding Sequence^b^
*rlgA*	Putative resolvase	2.8	AAAATTAAAATCGTTGGC
*map^c^*	Methionine aminopeptidase	2.6	AAATTGAGAATCATTCTG
*rpsB*	30S ribosomal subunit protein S2	4.0	AAATTGAGAATCATTCTG
*yajC*	Tranlocase protein, IISP family	3.2	GTAATGCAAAGCATAAAA
*nrdR^c^*	Putative transcriptional regulator	2.5	GAAACGGTAAAAATTACC
*sucC*	Succinyl-CoA synthetase, beta subunit	4.1	CTAAAGATAACGATTACC
*cmk*	Cytidine monophosphate kinase	2.7	AAAAAGTAAATCATTGTC
*STM1013*	Gifsy-2 prophage, regulatory protein	2.8	AAAATCAAAATCAGTAAC
*STM1133^c^*	Putative dehydrogenase	-4.2	ATAATGAGTAGAATTGTT
*nth^c^*	Endonuclease III	2.9	GAAAAGCGTACCATTCCC
*ldhA^c^*	Fermentative D-lactate dehydrogenase	-4.0	AATATGCTTAAAATTATC
*ynaF^c^*	Putative universal stress protein	-37.3	GAAATAGATATAATTTAT
*hns*	Histone like protein	3.1	ACAATGCTTATCATCACC
*STM1795^c^*	Homolog of glutamic dehydrogenase	5.8	AAAAAGATAAAAATAACC
*STM2186*	Putative glutamate synthase	-8.8	AAATTGAGAATAGTTATT
*eutC^c^*	Ethanolamine ammonia lyase	-4.1	ATAATGCCCATCGTTTCC
*eutB^c^*	Ethanolamine ammonia lyase	-3.2	AAACTGATAAACATTGCC
*yffB^c^*	Putative glutaredoxin	2.6	GAAATTCGAATAAATAAT
*iroN^c^*	TonB-dependent siderophore receptor	9.1	CTAATGATAATAATTATC
*yggU^c^*	Cytoplasmic protein	3.5	ATAACGCTAAGAATAAAC
*STM3600^c^*	Putative sugar kinase	-6.8	CTGATGCTCATCATTATT
*STM3690*	Putative lipoprotein	-4.2	ATAAACATTATAATTATA
*rpoZ^c^*	RNA polymerase, omega subunit	3.9	AATAAGATAATCATATTC
*udp^c^*	Uridine phosphorylase	-5.4	CAATAAATAATCAATATC
*yjcD^c^*	Putative xanthine/uracil permease	2.8	AAAAAGCAAACGATTATC
*dcuA*	Anaerobic dicarboxylate transport protein	-5.8	CAAATAACAACAATTTAA

^a ^Ratio of mRNA, Δ*fur*/14028s

^b ^Predicted Fur binding site located within -400 to +50 bp relative to ATG

^c ^Indicates the predicted Fur binding site is located on the reverse strand

#### a. Fur as a repressor

Genes associated with metal homeostasis were up-regulated in Δ*fur*. These included the well characterized genes/operons involved in iron homeostasis (i.e., *entABEC*, *iroBCDE*, *iroN*, *fes*, *tonB*, *fepA*, *bfr*, *bfd)*, Mn^2+ ^transport genes (i.e., *sitABC*), and copper resistance (i.e., *cutC*) [58-65] (Additional file 2: Table S2).

Expressions of genes involved in xylose metabolism (xylBR) were increased 3.7 and 2.9-fold, respectively, in Δ*fur *relative to the WT (Additional file 2: Table S2). In addition, the glycolytic genes *pfkA *and *gpmA *were 3.3-and 5.6-fold higher in Δ*fur*, respectively (Additional file 2: Table S2). Two genes, *STM1586 *(coding for a putative periplasmic protein) and *sitA *were up-regulated 76.1 and 53.8-fold, respectively, in Δ*fur *(Additional file 2: Table S2). These two genes exhibited the highest differential expression in Δ*fur*. Intriguingly, the microarray data showed that the gene for adenloysuccinate synthetase (*purA*), which is required for adenosine 5' monophosphate synthesis, was up-regulated 3.5-fold in Δ*fur*. Incidentally, *purA *mutants are known to be highly attenuated and have been used in developing *in vivo *expression technology (IVET) to detect promoters activated during *S*. Typhimurium infection [66,67].

Transcription of the cytochrome-o ubiquinol oxidase operon (*cyoABCDE*) and the high affinity cytochrome-d terminal oxidase genes (*cydAB*) was repressed by Fur (Additional file 2: Table S2). Interestingly, aerobic expression of *cydAB *is repressed by H-NS, which is relieved by the response regulator ArcA [68]. In addition, we detected increased expression of *hns *in Δ*fur *(Additional file 2: Table S2), and earlier work detected *in vivo *binding of Fur to the upstream region of *hns *[29]; this strongly indicates that Fur directly represses *hns *under anaerobic conditions. How or if H-NS may interact in the anaerobic regulation of *cydAB *under our conditions is unknown, since the repression of *cydAB *by H-NS does not appear to occur under anaerobic conditions [68].

Genes associated with DNA repair and purine metabolism (*nrdAB*, *nth*, *recA*, and *nei*) were repressed by Fur under anaerobic conditions (Additional file 2: Table S2), thus implicating Fur as a regulator of DNA repair and *de novo *synthesis. Fur was found to repress *ydiE *(*STM1346*) and a putative Fur binding site was found upstream of the start codon, where the expression of the gene was 7.4-fold higher in the mutant than in the wild-type (Additional file 2: Table S2). In *Yersinia enterocolitica*, YdiE has a conserved HemP (COG4256) domain, and is encoded within the hemin uptake operon [69]. Although *S*. Typhimurium is not known to utilize host's heme, previous work has established a Fur binding site upstream of *ydiE *and *hemP *in *S*. Typhimurium and *Y. enterocolitica*, respectively [16,69]. This indicates that our bioinformatic analyses indeed agree with experimentally identified Fur binding sites.

#### b. Fur as an activator

Anaerobic transcription of the fumarate reductase (*frdABD*) operon and the aspartase gene (*aspA*) was significantly lower in Δ*fur *(i.e., Fur is serving as an activator); however, the genes coding for the alpha and beta subunits of succinyl-CoA synthetase (*sucCD*) were up-regulated 4.1 and 2.7-fold, respectively (Additional file 2: Table S2). These genes (i.e., *frdABD*, *aspA*, *sucCD*) and *fumAB *(fumarate hydratase) are members of the reductive branch of the TCA cycle. We assayed for fumarate reductase (FRD) in cell-free extracts from anaerobic cultures and found that Fur is required for the anaerobic transcription and activity of FRD in *S*. Typhimurium (Additional file 3: Table S3). In *E. coli*, the transport of C_4_-dicarboxylates occurs via two seemingly redundant genes encoded by *dcuA *and *dcuB *[70]. In the present study, the *dcuB-fumB *operon was unaffected by Fur, while the *aspA-dcuA *operon was significantly down regulated in Δ*fur *and both genes contained a putative Fur box 5' of the start codon (Additional file 2: Table S2).

Genes involved in anaerobic respiration (*dmsABC*) and ethanolamine utilization (*eutSPQTDMEJGHABCLK*) were activated by Fur (Additional file 2: Table S2). The mechanism for reduced expression of *dmsABC *is unclear. Ethanolamine is a significant source of carbon and nitrogen during *Salmonella *infection [71].

One metabolic pathway that appears impacted by Fur is that required for glycerol metabolism. The genes for glycerol metabolism are located throughout the genome. For instance, *glpQT *and *glpABC *are divergently transcribed in two predicted operons. All of these genes were significantly down regulated in Δ*fur *(Additional file 2: Table S2). Furthermore, *glpD*, and *glpKF *were all down regulated in Δ*fur *(Additional file 2: Table S2). The down-regulation of these genes suggests that the Δ*fur *strain may be unable to utilize glycerol or transport glycerol- 3 phosphate. The mechanism of this regulation is unclear, but the absence of Fur binding sites in the promoters of any of these genes suggests an indirect mode of regulation. The contribution of glycerol metabolism to infection is unknown.

Another metabolic pathway, the *tdc *operon (required for the anaerobic transport and metabolism of L-threonine and L-serine [72,73]) was activated by Fur. The genes in this operon (*tdcBCDEG*) are activated by *tdcA *[74]. TdcA is a member of the LysR family of transcriptional activators [75]. Our data showed that the expression of all genes in this operon, *tdcABCDEG*, was significantly down-regulated in Δ*fur *(Additional file 2: Table S2). However, a Fur binding site was not identified in the promoters of any of the genes in the *tdc operon*, suggesting its indirect regulation by Fur. Importantly, H-NS is known to directly bind and repress this operon [31,76]. Therefore, the increased expression of *hns *in Δ*fur *(Additional file 2: Table S2), may account for the observed effect of Fur on the *tdc *operon. Mutations in the *tdc *operon have been shown to reduce invasion and virulence in *S*. Typhimurium [77,78]. In addition to the reduced expression of the *eut *operon, the reduced expression of the *tdc *operon and *hilA *may contribute to the observed attenuation of the Δ*fur *strain of *S*. Typhimurium [29,79].

### Role of Fur in regulation of antioxidant genes

Reactive oxygen and nitrogen species (ROS and RNS, respectively) are important host defense responses during bacterial infection. Our array data (Additional file 2: Table S2) revealed differential regulation of some important antioxidant genes whose products are essential for protecting the cells against ROS and RNS (i.e., superoxide dismutases, ferritin-like protein, and flavohemoglobin). Therefore, we decided to study the expression of these genes in greater detail.

#### a. Regulation of *sodA *and *sodB*

**Figure 3:**
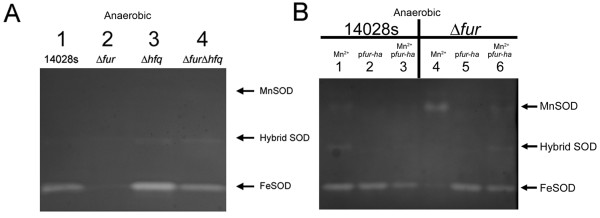
**Effects of Fur, Hfq, and manganese on the activity of superoxide dismutases**. **(A)** Effects of Fur and Hfq - Cell-free extracts from anaerobically grown cultures (14028s, Δ*fur*, Δ*hfq*, and Δ*furΔhfq*) were prepared as described in the Methods. Equal protein (125 μg/ml) was loaded and following electrophoresis the gel was stained for SOD activity. Lane 1 - 14028s; lane 2 - Δ*fur*; lane 3 - Δ*hfq*; lane 4 - Δ*furΔhfq*. **(B) **Effects of Fur and MnCl_2 _- Cell-free extracts were prepared from anaerobically grown cultures as in (A) except that 1 mM MnCl_2 _was added to the media. Equal protein (125 μg/lane) was loaded, elecrophoresed, and stained for SOD as in (A). Lane 1, 14028s + MnCl_2; _lane 2, 14028s + p*fur-ha*; lane 3, 14028s + MnCl_2 _+ p*fur-ha*; lane 4, Δ*fur *+ MnCl_2; _lane 5, Δ*fur *+ p*fur-ha*; lane 6, Δ*fur *+ MnCl_2 _+ p*fur-ha*.

There is plethora of information about the regulation of *sodA and sodB *in *E. coli *[80-85], but there is little knowledge about the regulation of these genes in *S*. Typhimurium [86]. In the present study, the microarray data showed that the anaerobic expression of *sodA *and *sodB *in Δ*fur *was > 9-fold higher and > 3-fold lower, respectively, than in the parent WT strain (Additional file 2: Table S2). SodA (MnSOD) and SodB (FeSOD) are the cytosolic superoxide dismutases of *S*. Typhimurium and they require the cofactors manganese and iron, respectively. These SODs are homodimers, and are fully functional when metalated with the appropriate metals (i.e., manganese for SodA and iron for SodB). However, a heterodimer consisting of SodA(Mn)/SodB(Fe) can still exhibit SOD activity, albeit at a reduced level compared to the homodimer [87]. Thus, in order to see an active hybrid SOD, both SodA and SodB must be expressed. Data in Figure 3A demonstrated that, as in anaerobic *E. coli*, the WT strain (Lane 1) lacked the activity of both Mn- and Hybrid-SODs, but possessed an active FeSOD. However, Δ*fur *(Figure 3A - Lane 2) was devoid of all three SOD-isozymes. The lack of FeSOD in Δ*fur *was of no surprise, as previous studies in *E. coli *[83,84] have established that Fur is indirectly required for the translation of *sodB *via its repression of the small RNA, *ryhB*, which works in conjunction with the RNA chaperon protein, Hfq [88,89]. Indeed, a strain harboring deletions in both Fur and Hfq (Δ*fur*Δ*hfq*) resulted in restoration of SodB activity (Figure 3A - Lane 4). Furthermore, the high degree of sequence identity in the promoter and the gene sequence of *ryhB *of *E. coli *with the two *ryhB-*like small RNAs, *rfrA *and *rfr *of *S*. Typhimurium [39], suggested that the regulation of *sodB *in *S*. Typhimurium is similar to that reported in *E. coli *[88,89]. Interestingly, expression of the hybrid SOD appears up-regulated in Δ*hfq *and Δ*fur*Δ*hfq *(Figure 3A - Lane 3 and 4). The reason for this is unclear, but may be due to the activation of the Hfq-binding small RNA (*fnrS*) by Fnr, which subsequently represses the expression of *sodA *[90,91].

The WT strain of *S*. Typhimurium possessed neither an active SodA (MnSOD) nor the hybrid enzyme (SodA/SodB), which is not surprising since this is normally the case in WT *E. coli *[92]. What was surprising is the lack of MnSOD activity in the anaerobic cell-free extracts from Δ*fur *(Figure 3A - Lane 2) in spite of the > 9-fold increase in the transcription of *sodA *(Additional file 2: Table S2). Therefore, we reasoned that the increased intracellular concentration of free iron in Δ*fur *[93] could result in competition of iron with manganese for the active site of SodA. This would lead to the formation of a non-active form of the enzyme, i.e., SodA-Fe instead of the active SodA-Mn (MnSOD). Analysis of total iron and manganese concentrations in our media showed that it contained ~40-fold more iron than manganese (i.e., ~7.5 μM iron *vs*. ~0.2 μM manganese). Additionally, the manganese content of anaerobic cultures of the parent strain and of the Δ*fur *strain were low, 0.09 ± 0.01 and 0.08 ± 0.04 μmoles manganese per gram of dry weight, respectively. Therefore, we supplemented the growth media with 1 mM MnCl_2 _and determined the SOD activities (Figure 3B). If our reasoning was correct, we expected that excess Mn^2+ ^added to the growth media would reveal increased MnSOD activity in Δ*fur*. Indeed, this was the case, as a dramatic increase in MnSOD was observed in Δ*fur*, but not in the parent strain (Figure 3B - lanes 1 *vs*.4). Also, cultures grown in presence of 1 mM MnCl_2 _contained 47.2 ± 2.7 and 48.8 ± 2.0 μmoles of manganese per gram of dry weight for the parent strain and for Δ*fur*, respectively. Altered MnSOD activity in Δ*fur *was due entirely to the lack of a functional *fur *gene since the introduction of a plasmid carrying the *fur *gene (i.e., p*fur-ha*) diminished MnSOD activity to that of the parent strain (Figure 3B - Lane 1 and 6). In addition, the plasmid p*fur-ha *restored FeSOD activity (Figure 3A - lane 5) as well as the phenotypic appearance of the WT strain observed on a Tris buffered chrome azurol agar plates (CAS plates) [94] containing 0.3% xylose [29]. These results indicated that increased transcription of *sodA *in Δ*fur *did not result in a corresponding increased MnSOD activity due to the excess intracellular free iron and that the addition of Mn^2+ ^negated this effect. On the other hand, the inclusion of excess Mn^2+ ^in the growth medium of the parent strain did not increase MnSOD activity, which indicated that Mn^2+ ^was not a signal for *sodA *induction. Furthermore, these findings demonstrated an important aspect of metalloenzyme regulation, i.e., the availability of the correct cofactor has a profound impact on enzyme activity.

#### b. Regulation of ftnB

**Figure 4:**
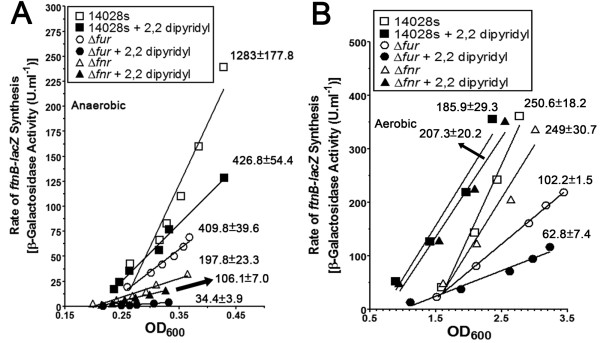
**Effects of Fur, Fnr and iron chelation on transcription of *ftnB***. Transcriptional *ftnB-lacZ *activity was determined in 14028s (squares), Δ*fur *(circles), and Δ*fnr *(triangles) under **(A) **anaerobic, and **(B) **aerobic conditions in LB-MOPS-X media without (open symbols) and with (closed symbols) 200 μM of 2, 2' dipyridyl. β-galactosidase assay was conducted throughout the growth of the culture and activity is presented in the form of differential plots with representative data shown in **(A) **and **(B)**. Best-fit lines, calculated as described in the Methods, are shown in **(A) **and **(B)**. For **(A) **and **(B)**, representative data are shown with the differential rate of synthesis (U/OD_600_) ± standard deviations from three independent experiments listed.

Microarray data (Additional file 2: Table S2) revealed a 7-fold reduction in the expression of *ftnB *in Δ*fur *as compared to the parent strain. The expression of *ftnB *was shown to be activated by Fnr [21]. Therefore, we used a chromosomal *ftnB-lacZ *transcriptional fusion in Δ*fur *and in Δ*fnr *genetic backgrounds to determine the contribution of each regulator in the expression of *ftnB*. The deletion of *fur *reduced the aerobic rate of synthesis of the reporter gene by > 2-fold compared to the parent strain (Figure 4A). 2, 2' dipyridyl (dip) reduced the rate of synthesis of the reporter gene in aerobic conditions (Figure 4A). Although induction of the reporter fusion occurred earlier in the growth phase with dip treated cultures, the rate of synthesis was reduced compared to untreated parent strain. This indicates inhibition by dip (Figure 4A). As expected, the oxygen sensitive regulator Fnr did not impact regulation of *ftnB *in aerobic conditions (Figure 4A). This indicated that Fur is required for *ftnB *expression, independent of Fnr. Data in Figure 4B show that the absence of *fur *resulted in a 2-fold reduction in the rate of synthesis (U/OD_600_) of *ftnB-lacZ *under anaerobic conditions. Furthermore, the ferrous iron chelator, dip, reduced the rate of anaerobic synthesis of *ftnB-lacZ *in the WT strain by > 2-fold (Figure 4B). In Δ*fur*, the rate of synthesis was further reduced (> 10-fold) when compared to the WT parent strain treated with dip (Figure 4B). In addition, the rate of synthesis in the parent strain was greatest under anaerobic conditions due to the active roles of both Fnr and Fur (Figure 4). Collectively, full expression of *ftnB *is dependent on Fur in aerobic and anaerobic conditions, whereas Fnr is a strong activator in the absence of O_2_.

#### c. Regulation of hmpA

**Figure 5:**
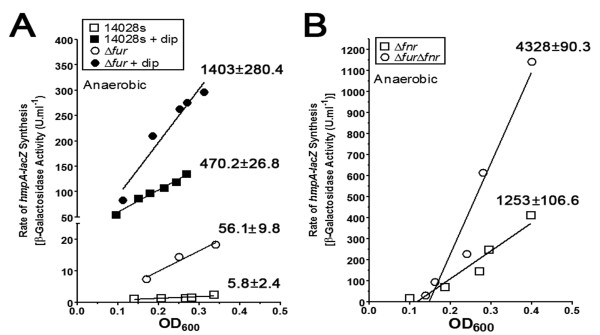
**Fur and Fnr control transcription of *hmpA***. **(A) **The transcriptional *hmpA-lacZ *activity was determined in 14028s and Δ*fur *under anaerobic conditions. The iron chelator 2, 2' dipyridyl (dip) was used at 200 μM; and **(B) **β-galactosidase activity was measured in Δ*fnr *and Δ*furΔfnr *backgrounds under anaerobic conditions - the best-fit lines are shown. For **(A) **and **(B) **representative data are shown with the differential rate of synthesis (U/OD_600_) ± standard deviations from three independent experiments listed.

The gene coding for the flavohemoglobin (*hmpA*), a NO^· ^detoxifying protein [95-98], was differentially expressed in Δ*fur *(Additional file 2: Table S2). Expression of *hmpA *is repressed by Fnr and another DNA binding protein that contains an iron sulfur cluster, NsrR [21,95-97,99]. Repression of *hmpA *by two regulators that are sensitive to RNS allows derepression of this gene under conditions of increased RNS. Indeed, regulation of *hmpA-lacZ *was induced ~80-fold by the nitrosating agent sodium nitroprusside in aerobic conditions (B. Troxell and H.M. Hassan, unpublished data). Under anaerobic conditions, *hmpA *was up-regulated 4-fold in Δ*fur*. Thus, we examined its anaerobic regulation with a chromosomal *hmpA-lacZ *transcriptional fusion. Figure 5 shows that the WT exhibited very little expression of *hmpA-lacZ *under anaerobic conditions (Figure 5A); suggesting regulation may be oxygen dependent. Indeed, expression was ~14-fold higher under aerobic conditions than anaerobic conditions (B. Troxell and H.M. Hassan, unpublished data). However, the addition of the iron chelator, dip, resulted in an increased rate of synthesis ~81-fold (Figure 5A). The increased expression of *hmpA-lacZ *by the addition of dip could have been due to inactivation of Fnr, Fur, and/or NsrR. We narrowed our focus to the roles of Fur and Fnr in regulation of this gene. In Δ*fur*, the reporter activity was up-regulated > 9-fold (Figure 5A), which confirmed the microarray data. The addition of dip increased the rate of synthesis by 25-fold in Δ*fur*. One known repressor of *hmpA *is Fnr [21,95-97]. Therefore, we combined the *fur *and the *fnr *deletions (Δ*fur*Δ*fnr*) in the *hmpA-lacZ *background to determine the role of Fur and Fnr in the regulation of *hmpA*. Deletion of *fnr *increased the rate of *hmpA-lacZ *synthesis by 216-fold as compared to the parent strain (Figure 5B). The synthesis of *hmpA-lacZ *in the Δ*fnr *mutant background was similar to that seen in the Δ*fur *treated with dip (i.e., 1253 ± 107 and 1403 ± 280 - U/OD_600_). The lack of an obvious Fur binding motif upstream of *hmpA *indicates that reporter activity seen in Δ*fur *was likely indirect. The combined deletion of *fur *and *fnr *in the *hmpA-lacZ *strain increased the rate of synthesis 746-fold as compared to the WT strain (i.e., 4328 ± 90 *vs*. 5.8 ± 2.4 - U/OD_600_) (Figure 5). Thus, the rate of synthesis of *hmpA-lacZ *in Δ*furΔfnr *was ~3.5-fold higher than the rate of synthesis in Δ*fnr *(i.e., 4328 ± 90 *vs*. 1253 ± 107 - U/OD_600_). Since we did not identify a discernable Fur binding site in *hmpA*, the fact that there is no published report showing Fur binding to the regulatory region of *hmpA*, and that the expression of *hmpA-lacZ *in Δ*furΔfnr *was ~3.5-fold higher than in Δ*fnr *demonstrates that under anaerobic conditions, Fur is indirectly regulating *hmpA-lacZ *independent of Fnr.

### Identification of new Fur targets

Table 3 shows genes differentially regulated in Δ*fur *that contain a putative Fur binding site located within -400 to +50 nucleotides relative to the translational start site. The putative translocase subunit, *yajC*, was up-regulated 3.2-fold in Δ*fur*. This gene is predicted to be in the Sec-dependent pathway of protein export. At least one other gene of the Sec-dependent pathway of protein export was up-regulated in Δ*fur*, *secY*. This gene, s*ecY*, is a direct target of Fur regulation in *Neisseria meningitides *[100,101]. Indeed, we detected a putative Fur binding site upstream of *secY *(Additional file 2: Table S2). The role of *yajC *during infection is unknown, but our results suggest Fur controls Sec-dependent protein secretion.

NrdR is a global transcriptional regulator that controls expression of oxygen-dependent and independent ribonucleotide reductases [102-104]. Expression of *nrdR *was up-regulated in Δ*fur *and a putative Fur binding site was identified. Although, deletion of *fur *results in up-regulation of *nrdHIEF *[105], a class Ib ribonucleotide reductase, we did not detect increased expression of this operon in our conditions. However, we did detect up-regulation of the class Ia ribonucleotide reductase, *nrdAB*, in Δ*fur *(Additional file 2: Table S2). The class III oxygen sensitive ribonucleotide reductase, encoded by *nrdDG*, is encoded in an operon. Expression of *nrdD*, the first gene of this operon, was down-regulated in Δ*fur *2.5-fold. (Additional file 2: Table S2). Our data indicate that Fur controls the class Ib and III ribonucleotide reductases, either directly or indirectly, under anaerobic conditions.

A putative dehydrogenase (*STM1133*) was down-regulated 4.2-fold in the Δ*fur *(Table 3). This gene contains a putative Fur binding site on the reverse DNA strand. *STM1133 *is the final gene in an apparent four gene operon of unknown function (*STM1130-1133*). The first gene of this operon, *STM1130*, was also down-regulated 7.9-fold in Δ*fur *(Additional file 2: Table S2); however, a Fur binding site was not identified upstream of *STM1130*. Interestingly, this operon is composed of the putative N-acetylneuraminic acid mutarotase (*STM1130*), a putative outer membrane protein (*STM1131*), a putative sialic acid transporter (*STM1132*), and a putative NAD (P) binding dehydrogenase (*STM1133*). Thus, our results suggest Fur controls at least a portion of this operon that may be localized to the bacterial membrane. The importance of these genes during infection is unknown.

Several putative genes appear to be under direct control of Fur. Genes that exhibited reduced expression in Δ*fur *were the putative universal stress protein encoded by *ynaF*, the putative glutamate synthase (*STM2186*), the putative sugar kinase (*STM3600*), and the putative lipoprotein (*STM3690*). The putative Fur binding site for *ynaF *and *STM3600 *is located on the reverse strand for these genes. The mechanism of Fur activation of these putative genes is unknown. In addition, several putative genes exhibited up-regulation in Δ*fur*. A putative glutamic dehydrogenase (*STM1795*), a putative glutaredoxin (*yffB*), and a putative protein (*yggU*), were all up-regulated in Δ*fur*. Interestingly, *yffB *is predicted to be a glutathione-dependent thiol reductase. The contribution of these genes to infection is unknown.

The TonB siderophore receptor gene, *iroN*, was up-regulated 9.1-fold in Δ*fur*. Despite the widespread study of siderophores (salmochelins) in *Salmonella *virulence, we were unable to find any published report that Fur represses *iroN*. Although Fur repression of the *iroBCDE *loci is known [59], *iroN *is encoded downstream of this operon and is transcribed in the opposite orientation. Our results confirm the prediction by Baumler e*t al *that *iroN *is regulated by Fur [58].

## Discussion

Iron is essential in most pathogenic bacteria, which compete rigorously with the host for this element. *S*. Typhimurium is no exception. The 17-kDa transcriptional regulator, Fur, plays an important role in bacterial iron homeostasis. Although publications of Fur regulation in *E. coli *and other bacteria are numerous, this is the first report on the global role of Fur in anaerobically grown *S*. Typhimurium. Indeed, anaerobic metabolism has been shown to be important for pathogens and pathogenesis [21-24,29].

In this study, we found that, under anaerobic conditions, Fur directly or indirectly affected the expression of 298 genes (Additional file 2: Table S2). A putative Fur binding motif was identified in 49 genes (Table 4. column #1). Also, Table 4 shows evidence of published data demonstrating the role of Fur in their regulation (column #3) and published experimental evidence for Fur binding to the regulatory region of these genes (column #4). The role of other co-regulators is also shown (Table 4, column #5). Interestingly, twelve of the 49 genes contained the binding motifs for both Fnr and Fur (Additional file 4: Table S4).

**Table 4:** Comparison of Differentially Expressed Genes in Δ*fur *That Contain a Putative Fur Binding Site with Confirmed Data of Fur Regulation from other Studies and the Possible Involvement of other Transcription Regulators

Genes Regulated by Fur and containing a putative Fur motif^a^	Fold Change^b^	Published Evidence of Fur Regulation [Ref.]	Published Evidence of Fur Binding [Ref.]^c^	Published Evidence of Control By Other Regulators [Ref]^d^
*rlgA*	2.8	No	No	
*map*	2.6	No	No	
*rpsB*	4.0	No	No	
*yajC*	3.2	No	No	
*nrdR*	2.5	No	No	
*cyoE*	3.1	Yes [12]	No	Fnr [21]
*cyoD*	7.1	Yes [12]	No	Fnr [21]
*cyoB*	8.2	Yes [12]	No	Fnr [21]
*cyoA*	3.2	Yes [12]	No	Fnr [21]
*fepA*	10.7	Yes [12,15,16,126-129]	Yes [128,129]	
*fes*	39.8	Yes [12,16,127-129]	Yes [128,129]	
*entC*	6.8	Yes [12,15,130]	Yes [130]	
*sucC*	4.1	No	No	Fnr [21]
*gpmA*	5.6	Yes [12]	No	
*cmk*	2.7	No	No	
*STM1013*	2.8	No	No	
*STM1133*	-4.2	No	No	Fnr [21]
*ydiE*	7.4	Yes [12,15]	No	Rcs [131]
*nth*	2.9	No	No	
*STM1586*	76.1	Yes [15]	No	
*ldhA*	-4.0	No	No	Fnr [21]
*ynaF*	-37.3	No	No	Fnr [21]
*tonB*	11.4	Yes [12,15]	Yes [132]	
*hns*	3.1	Yes [29]	Yes [29]	
*STM1795*	5.8	No	No	Fnr [21]
*STM2186*	-8.8	No	No	Fnr [21]
*cirA*	4.0	Yes [12,15]	Yes [133]	
*eutC*	-4.1	No	No	Fnr [21]
*eutB*	-3.2	No	No	Fnr [21]
*yffB*	2.6	No	No	
*iroB*	4.6	Yes [15,59]	No	
*iroN*	9.1	No	No	
*sitA*	53.8	Yes [15,46,61,134-138]	No	MntR [61]
*yggU*	3.5	No	No	
*yqjH*	3.8	Yes [12]	No	
*secY*	4.0	Yes [101]	Yes [100]	
*bfr*	3.2	Yes [14,79,88]	No	
*bfd*	5.9	Yes [12,14,15]	No	
*feoB*	11.8	Yes[12,14,63,134,139,140]	No	ArcA and Fnr [141]
*STM3600*	-6.8	No	No	Fnr [21]
*STM3690*	-4.2	No	No	Fnr [21]
*rpoZ*	3.9	No	No	
*udp*	-5.4	No	No	IscS [142]
*sodA*	9.1	Yes [14,55,82,88,143-148]	Yes [85,146,148]	Fnr, ArcA, IHF, SoxRS [53,81]
*yjcD*	2.8	No	No	
*dcuA*	-5.8	No	No	
*aspA*	-3.6	Yes [13,15]	No	NarL[149,150] ArcA [151]
*ytfE*	10.0	Yes [13]	No	NsrR [99]
*fhuF*	8.5	Yes [12,13,15]	Yes [11,152,153]	

^a ^Genes from the present study that are regulated by Fur and possess a putative Fur-binding motif

^b^Fold change of expression in Δ*fur *relative to the wt 14028s

^c ^Evidence of direct Fur binding the regulatory region of the gene

^d ^Regulation by other transcription factors besides Fur

**Figure 6:**
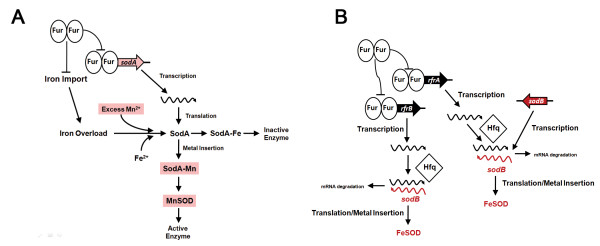
**Role of Fur in the transcriptional, translational and post-translational regulation of *sodA *and *sodB***. **(A) **Repression of *sodA *by Fur is depicted in addition to the role of Fur in iron homeostasis. Iron is known to bind to the active site of MnSODs that leads to inactivation of the enzyme [106,124]. Increased expression of MnSOD was detected only when excess Mn^2+ ^was added to the media in order to out compete the Fe^2+^. Deletion of *fur *under iron replete conditions results in increase transcription of *sodA*, but incorportation of Fe^2+ ^into the active site of SodA resulting in SodA-Fe and an inactive enzyme. Addition of excess Mn^2+ ^to the culture media can out compete Fe^2+ ^for the active site of SodA resulting in SodA-Mn and an active enzyme. **(B) **Indirect regulation of SodB by Fur in *S*. Typhimurium. The small RNAs *rfrA *and *rfrB *of *S*. Typhimurium are likely to function as their homolog *ryhB *in *E. coli *in regards to SodB regulation [88]. Our data confirms the Hfq-dependent function of reduced FeSOD activity in Δ*fur*. Previous work confirmed the role of Hfq and Fur in SodB expression [39]. Deletion of *fur *results in increased transcription of the sRNAs (*rfrA *and *rfrB*) that can pair with mRNA of *sodB *in an Hfq-dependent fashion and result in the degradation of *sodB *mRNA. However, a combined deletion of *hfq *in Δ*fur *results in loss of *rfrAB*-mediated degradation of *sodB*, and results in the synthesis of SodB protein that gets activated to FeSOD in the presence of Fe^2+^.

The appropriate metal cofactor was shown to be essential for detection of MnSOD activity, in spite of the 9-fold increase in *sodA *transcript for Δ*fur*. Therefore, genetic backgrounds that alter the steady-state [Mn^2+^] or its competitor [Fe^2+^] may have dramatic effects on MnSOD activity. Indeed, we were only able to discern the role of Fur in *sodA *and MnSOD expression with the addition of excess MnCl_2 _to the growth media. These data are summarized in Figure 6, which depicts the transcriptional, translational, and post-translational role of Fur in *sodA *and *sodB*. This implies that disruption of iron homeostasis is likely to have a two-pronged effect, increase in Fenton chemistry and a decrease in MnSOD activity due to iron overload. It appears that the inhibition of MnSOD by iron is evolutionarily conserved. Thus, the mitochondrial Mn^2+^-cofactored SOD2 has been shown to be inactivated in a similar manner when iron homeostasis was disrupted in yeast [106]. In addition, supplementation of the medium with Mn^2+ ^reduced oxidative stress in a murine model of hemochromatosis [107]. It is unknown if this is due to enhanced MnSOD or if Mn^2+ ^supplementation reduces oxidative stress in other pathological states of altered iron homeostasis.

**Figure 7:**
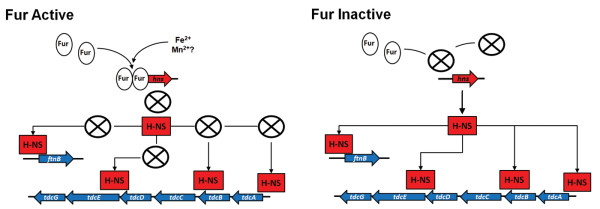
**Representation depicting the role of Fur and H-NS in the regulation of *ftnB *and the *tdc *operon**. H-NS confirmed binding sites and transcriptional repression [31] were compared with our microarray data and Fur repression of *hns *[29]. Collectively, the data indicate that Fur-dependent activation of *ftnB *and the *tdc *operon may be due to the increased expression of H-NS in Δ*fur*, which represses *ftnB *and the *tdc *operon. Thus, under Fur active conditions (left panel), *hns *is repressed by Fur thereby blocking H-NS repression of *ftnB *and the *tdc *operon (signified by the circle with an "X"). While under Fur inactive conditions (right panel), the overexpression of H-NS results in the repression of *ftnB *and the *tdc *operon under anaerobic conditions.

Our decision to further study *ftnB *and *hmpA *was due to our previous findings, where we found that *ftnB *and *hmpA *were activated and repressed by Fnr, respectively [21]. The Fnr-dependent expression of *ftnB *was apparent from the reduced activity in Δ*fnr *under anaerobic conditions, and the reduced activity in the WT strain in presence of oxygen. In addition, iron chelation and the deletion of *fur *reduced *ftnB *expression regardless of the oxygen tension. These results indicated that Fur controlled regulation of *ftnB *is independent of Fnr. Our results are in agreement with earlier work that demonstrated dependence of *ftnB *expression on Fur [15]. However, they are contrary to a previous report, which determined that Fur exhibited a repressive role on *ftnB *expression [79]. The reason for this discrepancy is unclear. It is evident from work reported herein and in a previous study in *E. coli *that *ftnB *exhibits a strong dependence on low O_2 _conditions [108]. Furthermore, the earlier study [108] determined that Fnr bound the promoter of *ftnB *in *E. coli *and that the Fnr binding site was further upstream than in known Fnr regulated genes. The same investigators [108], postulated that Fnr was unable to induce *ftnB *and that other regulators were required. However, we have determined that Fnr alone contributes to the activation of *ftnB *and that Fur is required for full induction of the gene, with Fnr exhibiting a more pronounced role. The lack of a predicted Fur binding site in *ftnB *indicated that Fur regulation was indirect. The following scenario is proposed to explain these findings and to suggest that the observed regulation of *ftnB *by Fur is mediated by the histone-like protein H-NS. First, the microarray data showed that Fur negatively regulates the expression of *hns *and has a predicted Fur binding site (Table 3). Second, we recently demonstrated that Fur binds upstream of *hns *in a metal dependent fashion [29]. Third, whole genome ChIP analysis demonstrated that H-NS binds to *ftnB *and the expression of *ftnB *is up-regulated in the absence of *hns *[31]. Fourth, the *tdc *operon is a known target for H-NS repression [31,76] and was significantly reduced in the absence of *fur*. Therefore, we propose that the positive regulation *ftnB *by Fur is mediated by the negative regulation of *hns *by Fur. Thus removal of Fur (i.e., as in Δ*fur*) results in repression of *ftnB *by H-NS (see Figure 7). A second possibility is reduced Fnr function (or an additional activator) in Δ*fur *since several Fnr regulated genes were differentially expressed in Δ*fur*. However, our data rule-out this possibility in *ftnB *regulation by showing the involvement of Fur in the regulation of *ftnB *under aerobic conditions, where Fnr is inactive.

H-NS controls diverse functions within the cell and forms complex structures when binding DNA that indicates a central role in DNA topology [109-113]. Similar to Fur, H-NS is a repressor of transcription [31,34,35,114]. This implies that genes controlled by H-NS are regulated by iron through Fur. This interaction also demonstrates interaction between two regulators (Fur and H-NS) functioning in highly conserved physiological events, regulating a potentially toxic, but needed metal and regulating foreign DNA in a concerted manner. Thus, our results provided additional insight into iron-dependent regulation of H-NS.

Another gene regulated by Fnr or Fur was the NO^· ^detoxifying flavohemoglobin protein encoded by the *hmpA*. This gene (*hmpA*) is repressed by Fnr and contained a putative Fnr binding site, but did not contain a predicted Fur binding site [21,95,96]. Previous work determined that Fur was a repressor of *hmpA *[115]. However, it was later revealed that the reporter fusion was to the Fur repressed *iroC *and not to the *hmpA *[116]. Additionally, a previous report did not reveal a role for Fur in regulation of *hmpA *[97], while two other studies found a modest effect of Fur on *hmpA *expression [98,117]. NsrR is another repressor of *hmpA *[97]. Thus, *hmpA *is repressed by two regulators that contain an iron-sulfur cluster. Despite contradictory reports, increased *hmpA *expression was detected in Δ*fur*. Our initial hypothesis was that this was due to reduced Fnr function in Δ*fur*. To support this hypothesis, we expected reporter activity to be similar in Δ*fnr *and Δ*furΔfnr *backgrounds. However, our results did not support this initial hypothesis since Δ*furΔfnr *exhibited ~3.5-fold increased expression compared to Δ*fnr*; indicating that Fur regulation was Fnr-independent.

A striking finding was the shared regulation of several genes by Fur and Fnr. Microarray and bioinformatic studies indicated that 12 of the 298 Fur-dependent genes contained a predicted binding site for both Fur and Fnr. Thus, these two global regulators may be directly involved in regulation of these 12 genes (Additional file 4: Table S4). The expression data indicated that Fur and Fnr cooperate in the regulation of these 12 genes. For instance, each gene was regulated in the same manner in Δ*fur *or Δ*fnr*; a gene activated by Fur was also activated by Fnr. Lastly, our investigations indicate that Fur indirectly regulates genes that are under control of Fnr or additional regulators with an iron sulfur cluster (i.e., *ftnB *and *hmpA*). Furthermore, the observed reduced expression of the ethanolamine operon, *frdABD*, and *dmsABC *in Δ*fur*, suggest altered regulation of operons induced under anaerobiosis (Additional file 2: Table S2). Thus, Fur is an activator of genes that are typically induced under anaerobic conditions. Ethanolamine utilization within the host is important for *S*. Typhimurium and the Gram-positive pathogen *Listeria monocytogenes *[118,119]. In addition, Fnr is an activator of the *frd *and *dms *operons, which are responsible for anaerobic utilization of fumarate and dimethyl sulfide as alternative electron acceptors, respectively [120-123]. Our study of the anaerobic expression of *hmpA *suggests that it is regulated by Fur, independent of Fnr. Clearly, these results suggest Fnr is functional in Δ*fur *and that Fur is regulating genes of anaerobic metabolism (*eut*, *frd*, and *dms *operons) through an unknown mechanism.

## Conclusions

We demonstrated that Fur is an activator of *ftnB *in *S*. Typhimurium, which is likely due to the de-repression of *hns *in Δ*fur*. The strong dependence of *ftnB *expression on O_2 _indicates that Fnr is crucial in its regulation. Additionally, we presented evidence that Fur indirectly controls *hmpA*, independent of Fnr. We determined that Fur represses *sodA *transcription, but is required for the maturation of SodA into an active enzyme, MnSOD. Finally, we identified new target genes regulated by Fur in *S*. Typhimurium, and our data support the increasing evidence of enhanced H-NS expression in Δ*fur*.

## Authors' contributions

All authors have read and approved this work. BT, RCF, HMH designed and conducted the experiments and contributed to the writing and editing of the manuscript. RCF conducted the microarrays, constructed the Fur Logo, and contributed to the editing of the manuscript. MM and SP constructed and provided the microarray slides and reviewed the manuscript. BT and HMH conceived the research idea, directed the research, and contributed to the writing and editing of the manuscript.

## Supplementary Material

Additional file 1 **Table S1. Primer table**. This file contains the sequence of primers used in this study. Click here for file

Additional file 2 **Table S2. Fur Regulated Genes**. This file contains the genes that were differentially expressed between 14028s and Δ*fur *under anaerobic conditions. Click here for file

Additional file 3 **Table S3. Fumarate reductase activity under anaerobic conditions**. This file contains the specific activity of fumarate reductase in cell-free extracts isolated from 14028s and Δ*fur *under anaerobic conditions. Click here for file

Additional file 4 **Table S4. Genes regulated by Fur and Fnr under anaerobiosis and contain putative binding sites for both regulators**. This file contains genes that were differentially expressed in 14028s, Δ*fur*, and the *fnr*, which contain a putative binding site for Fur and for Fnr. Click here for file
