# Supplementary material for: The Fur regulon in anaerobically grown Salmonella enterica sv. Typhimurium: identification of new Fur targets
Source: BMC Microbiol. 2011 Oct 21;11:236. doi: 10.1186/1471-2180-11-236 (PMC3212961; doi:10.1186/1471-2180-11-236)
Supplement: Additional file 1 — Table S1. Primer table. This file contains the sequence of primers used in this study. [file 1471-2180-11-236-S1.PDF]

Table S1: Primers Used

Primer Name	Sequence	Relevance
FnrpKD3 fwd	AAAATTGACAAATATCAATTACGGCTTGAGCAGACCTATGGTGTAGGCTGGAGCTGCTTC	*fnr::cat*
FnrpKD3 rev	CGATATGGCAGAAGATAACATCAATGGTTTAGCTGACGTCATGGGAATTAGCCATGGTCC	*fnr::cat*
ftnBP1 fwd	CTAAGCGAATGGTGTTCTGAACACAGTTTAACAGGCACCGCTGGAGCTGCTTCGAAGTT	*ftnB::kan*
ftnBP2 rev	ATGCTGATCGGTTTGCGCCAGGCATAGCCCTGCTCGTTTTTCCGGGGATCCGTCGACCT	*ftnB::kan*
pCE36 rev	TCATCAACATTAAATGTGAGCGAG	Confirmation of *lacZ* fusions
