# Supplementary material for: The Fur regulon in anaerobically grown Salmonella enterica sv. Typhimurium: identification of new Fur targets
Source: BMC Microbiol. 2011 Oct 21;11:236. doi: 10.1186/1471-2180-11-236 (PMC3212961; doi:10.1186/1471-2180-11-236)
Supplement: Additional file 3 — Table S3. Fumarate reductase activity under anaerobic conditions. This file contains the specific activity of fumarate reductase in cell-free extracts isolated from 14028s and Δfur under anaerobic conditions. [file 1471-2180-11-236-S3.PDF]

Table S3 - FRD Activity from Anaerobic Samples

Strain	FRD[a] (Fold Change)
14028s	0.93 ± 0.15 (1)
Δ*fur*	0.2 ± 0.03 (- 4.7)

[a] Fumarate reductase activity is expressed as µmol of reduced benzyl viologen oxidized min^{-1} mg^{-1}.

Data shown are from 3 independent cultures ± SD.
