# Supplementary material for: The Fur regulon in anaerobically grown Salmonella enterica sv. Typhimurium: identification of new Fur targets
Source: BMC Microbiol. 2011 Oct 21;11:236. doi: 10.1186/1471-2180-11-236 (PMC3212961; doi:10.1186/1471-2180-11-236)
Supplement: Additional file 4 — Table S4. Genes regulated by Fur and Fnr under anaerobiosis and contain putative binding sites for both regulators. This file contains genes that were differentially expressed in 14028s, Δfur, and the fnr, which contain a putative binding site for Fur and for Fnr. [file 1471-2180-11-236-S4.PDF]

Table S4 - Genes regulated by Fur and Fnr under anaerobiosis and contained putative binding sites for both regulators

STM Number	STM Gene Name	Description	Fold Change[a]	Fold Change[b]
STM0439	*cyoE**	protohaeme IX farnesyltransferase (haeme O biosynthesis)	3.1	7.7
STM0440	*cyoD**	cytochrome o ubiquinol oxidase subunit IV	7.1	7.1
STM0442	*cyoB**	cytochrome o ubiquinol oxidase subunit I	8.2	5.0
STM0443	*cyoA**	cytochrome o ubiquinol oxidase subunit II	3.2	4.5
STM1133	*STM1133**	putative dehydrogenases and related proteins	-4.2	-5.6
STM1647	*ldhA**	fermentative D-lactate dehydrogenase, NAD-dependent	-4.0	-24.4
STM1652	*ynaF**	putative universal stress protein	-37.3	-116.2
STM1795	*STM1795**	putative homologue of glutamic dehyrogenase	5.8	5.3
STM2186	*STM2186**	putative NADPH-dependent glutamate synthase beta chain or related oxidoreductase	-8.8	-4.2
STM2458	*eutB**	ethanolamine ammonia-lyase, heavy chain	-3.2	-6.3
STM3600	*STM3600**	putative sugar kinases, ribokinase family	-6.8	-12.6
STM3690	*STM3690**	putative inner membrane lipoprotein	-4.2	-5.7

[a] Ratio of Δ*fur* /wild-type expression levels

[b] From [21].

* Indicate a putative Fur and Fnr binding site present in -400 +50 region 5' of start codon.
